# The structure of Toho1 β‐lactamase in complex with penicillin reveals the role of Tyr105 in substrate recognition

**DOI:** 10.1002/2211-5463.12132

**Published:** 2016-11-07

**Authors:** Patricia S. Langan, Venu Gopal Vandavasi, Kevin L. Weiss, Jonathan B. Cooper, Stephan L. Ginell, Leighton Coates

**Affiliations:** ^1^ Biology and Soft Matter DivisionOak Ridge National LaboratoryTNUSA; ^2^ Birkbeck University of LondonUK; ^3^ Structural Biology CenterArgonne National LaboratoryILUSA

**Keywords:** antibiotic resistance, antibiotics, enzyme, enzyme structure, X‐ray crystallography

## Abstract

The role of the conserved residue Tyr105 in class A β‐lactamases has been the subject of investigation using both structural studies and saturation mutagenesis. Both have shown that while it does not need to be strictly conserved for activity, it is important for substrate recognition. With this in mind we determined the crystal structure of Toho1 β‐lactamase at 15 K to 1.10 Å resolution in complex with penicillin. As expected a ring‐opened penicillin molecule bound to Ser70 the catalytic nucleophile, can clearly be seen in electron density in the active site. In addition to the trapped penicillin, however, are two additional intact ring‐closed penicillin molecules, captured by the enzyme through noncovalent interactions at the edge of the active site.

**Abbreviations**
ESBL: extended spectrum β lactamase
PBPs: penicillin binding proteins
PNM: ring‐opened penicillin molecule
PNN: ring‐closed penicillin molecule

Penicillin, a β‐lactam antibiotic, was first observed by Alexander Fleming in 1928 when he saw that mold growing on an accidentally contaminated Petri dish destroyed the bacteria surrounding it 1. This discovery changed the course of medical history. Unfortunately, however, resistant bacteria have been evolving alongside antibiotic agents long before their discovery by humans 2. Bacteria have obtained resistance to β‐lactam antibiotics in various ways, but most commonly by their expression of β‐lactamases. Despite evolving resistance to β‐lactam antibiotics they remain the most widely used class due to their low toxicity. This is due to the fact that they interact with bacterial penicillin binding proteins (PBPs) which are absent in humans. PBPs are enzymes involved in the construction of the bacterial cell wall. β‐lactams are able to form stable acyl‐enzyme complexes with PBPs, rendering them inactive, unable to maintain and construct the cell wall, ultimately causing cell lysis. As there is no human counterpart of this enzyme, and disruption of its activity is fatal to the bacterial cell, it is important that we continue to develop our understanding of how β‐lactam antibiotics are bound and inactivated by β‐lactamase enzymes 3, 4.

β‐lactamase enzymes are a family of hydrolytic enzymes expressed by resistant bacteria. They can be divided into four distinct classes (A–D) based on amino acid sequence identity 5. Classes A, C and D employ a catalytic serine residue to hydrolyze the β‐lactam molecule by formation and release of an acyl‐enzyme intermediate (Fig. 1, stage 3). Class B β‐lactamases utilize metal ions at the active site, enabling a nucleophilic hydroxide to break the β‐lactam bond. Typical class A β‐lactamases are TEM, SHV and the emergent extended‐spectrum β‐lactamase (ESBL) CTX‐M enzymes. These class A CTX‐M ESBLs are often found in clinical isolates associated with intra‐abdominal and urinary tract infections from highly virulent bacteria. Infection with bacteria that express CTX‐M ESBLs leads to treatment problems in many clinical settings and dramatically increases mortality rate due to their broad substrate profile. ESBLs exhibit increased hydrolytic activity against the first, second, and third generation extended‐spectrum cephalosporins and monobactams 5, 6, 7, 8, 9, 10, 11. The Toho‐1 β‐lactamase is a CTX‐M‐type ESBL on the basis of its amino acid sequence and its broad activity against the extended‐spectrum cephalosporins. Toho‐1 is composed of 262 amino acid residues and made up of two highly conserved domains (α/β and α). The active site cavity is formed at their interface.

**Figure 1:**
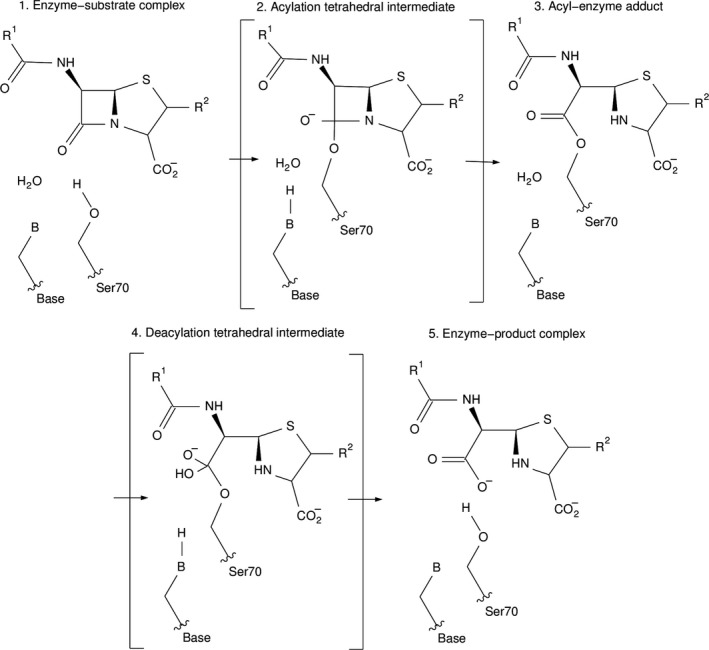
After substrate binding, Ser70 attacks the carbonyl carbon of the β‐lactam ring to form an acyl‐enzyme intermediate, which is then deacylated to liberate the inactivated antibiotic. Glu166 plays a vital role in the deacylation step (stages 3–5), by acting as the activating base of a hydrolytic water molecule. Mutating Glu166 halts the reaction at the acyl‐intermediate (stage 3), allowing this state to be characterized structurally. It has also been proposed that Glu166 acts as the catalytic base in the acylation step of the reaction (stages 1–3) by deprotonating the hydroxyl of Ser70 via a water molecule. This allows Ser70 to attack the carbonyl carbon of the β‐lactam ring.

Wild‐type β‐lactamases rapidly hydrolyze and release β‐lactam antibiotics, making it virtually impossible to trap the acyl‐enzyme intermediate for study. In class A β‐lactamase enzymes Glu166 activates the hydrolytic water required for deacylation of the tetrahedral intermediate. Therefore, mutating Glu166 to an alanine halts the reaction at the acyl‐enzyme adduct (Fig. 1, stage 3) enabling this state to be studied. The mutations R274N and R276N prevent crystal twinning and increase diffraction resolution 12 without dramatically affecting the kinetics of the enzyme 13. Tyr105 is a conserved active‐site residue throughout class A β‐lactamases and is located by the edge of the active site cavity. It has been shown that the Y105F mutant 14, 15 retains 52% catalytic efficiency toward benzyl penicillin compared to wild type. This rules out a critical role for the hydroxyl group of Tyr105 in enzyme activity, whilst indicating that it has a role in efficiency. It has previously been suggested that Tyr105 may stack with the benzyl ring of penicillins 16, 17 suggesting its participation in substrate positioning within the active site. However, to date this interaction between Tyr105 and the substrate molecule has never been shown or directly characterized.

## Materials and methods

### Expression and purification of recombinant protein

The Perdeuterated‐Toho‐1 E166A/R274N/R276N was purified and crystallized as described previously 18, 19, 20. Protein was produced to a high yield in a fully deuterated minimal medium using a fed‐batch fermentation protocol in an *Escherichia coli*‐based expression system 19, 21. This purification involves cell adaptation to deuterated minimal media, cell culture in a bioreactor, cell lysis and purification, and finally deuterium back exchange 19. After cell growth in minimal media, and harvesting, pellets were stored at −80 °C. For purification, pellets were thawed with lysis buffer (50 mm MES pH 6.5) and protease an inhibitor cocktail, at room temperature. Once thawed, the cells were lysed by sonication with a Branson Digital Sonifier (Emerson Industrial Automation, St. Louis, MO, USA). Lysate was then clarified by centrifugation at 34 000 ***g***, for 45 min at 4 °C. Supernatant was filtered through 0.45 μm syringe filter (GE Healthcare, Pittsburgh, PA, USA). Protein purification was achieved with an AKTA purifier system (GE Healthcare), at room temperature. A HiLoad SP Sepharose FF column (GE Healthcare) was equilibrated with Buffer A (20 mm MES, pH 6.5), and protein was loaded onto the column. A linear gradient of buffer B (20 mm MES pH 300 mm NaCl pH 6.5) was used to elute the protein at approximately 20–30 mm NaCl. Using UV absorbance, and SDS/PAGE gel analysis, fractions containing pure protein were selected and pooled. These fractions were concentrated using a 10 K MWCO Vivaspin 15R concentrator (Sartorius, Gottingen, Germany). Concentrated protein was loaded onto a Buffer‐A‐equilibrated 120‐ml HiPrep Sephacryl S‐100 HR gel filtration column (GE Healthcare). Using UV absorbance, and SDS/PAGE gel analysis, fractions containing pure protein were selected and pooled. These fractions were concentrated using a 10 K MWCO Vivaspin 15R concentrator (Sartorius). Buffer exchange was performed, and protein concentration measured using UV absorbance, before crystallization. We originally perdeuterated the protein for neutron studies 22 while also using it for several X‐ray studies.

### Crystallization

Crystals for X‐ray diffraction were grown at 20 °C via the batch crystallization method using 30 μL of 10 mg·mL^−1^ protein concentration in a solution containing 2.0 m ammonium sulfate and 0.1 m sodium citrate (pH 6.1). For ligand soaking, crystals were placed for 2–3 h in a reservoir solution containing 2.7 m ammonium sulfate, 0.1 m sodium citrate (pH 6.1), and 5.0 mm benzyl penicillin. The crystals were then placed momentarily in a reservoir solution containing a cryoprotectant (30% w/v trehalose) and subsequently flash‐frozen in liquid nitrogen.

### Data collection and refinement

Data were collected at 15 K on a protein crystal to beyond atomic resolution (1.10 Å) on the SBC‐CAT sector 19 beamline at the Advanced Photon Source. High‐resolution monochromatic data (0.67 Å) were collected, using a Cryo Industries of America (Manchester, NH, USA) cryocool helium cryostream at 15 K. The 15 K X‐ray data were processed using the xds package 23, scaled using scala 24 and refined with shelx 25 to convergence. All model building was done using the coot molecular graphics program 26. Figures were created using pymol 27, ligplot 28, and chemtool. The data reduction and refinement statistics for the structure are given in Table 1.

**Table 1:** Data collection and refinement statistics for the X‐ray diffraction data. Values for the highest resolution bin are given in parentheses

PDB accession code	5KMW
Unit‐cell parameters (Å)	*a* = 72.26, *b* = 72.26, *c* = 98.19 α = β = 90° γ = 120°
Space group	P3_2_21
No. of unique reflections	157 550
Resolution range (Å)	38.62–1.10 (1.16–1.10)
Multiplicity	5.5 (5.6)
*I*/σ(*I*)	5.6 (2.1)
*R*_merge_ (%)	9.3 (40.1)
*R*_pim_ (%)	3.8 (16.9)
Data completeness (%)	99.7 (98.6)
Crystallographic refinement	
*R*_factor_ (%)	11.52
*R*_free_ (%)	14.34
Ramachandran plot	
Outliers (%)	1.29
Favored (%)	96.78

This 15 K data collection temperature produced an average mean anisotropic displacement parameter for all protein main chain atoms of 8.50 and 12.43 Å^2^ for the side chain atoms. This is roughly a two‐fold reduction compared to similar data sets collected to the same resolution at 100 K using a standard 100 K nitrogen cryostream. These low displacement parameters allowed us to identify two intact ring‐closed benzyl penicillin molecules at the edge of the active site and enabled the direct visualization of previously unseen features of enzymatic substrate recognition involving the conserved residue Tyr105.

## Results and discussion

### Penicillin binding in the active site

**Figure 2:**
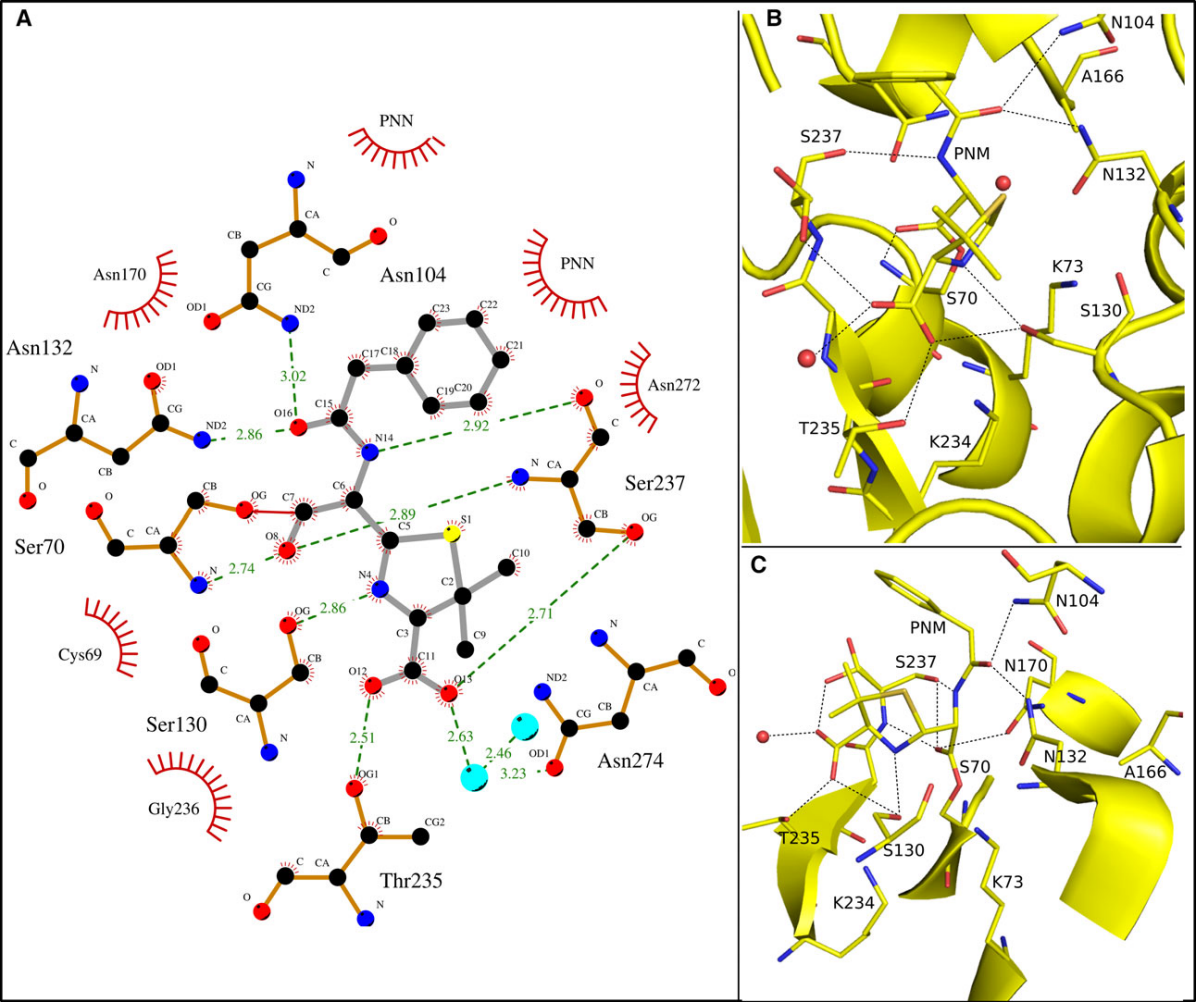
Hydrogen bonds and hydrophobic interactions between benzyl penicillin and E166A/R274N/R276N Toho1 β‐lactamase mutant, trapped in the acyl‐intermediate. (A) Carbon atoms are shown in black, nitrogen in blue, oxygen in red, and sulfur in yellow. Water molecules are indicated by turquoise spheres. The ring‐opened penicillin molecule (PNM) bonds are drawn as grey lines. Enzyme bonds are shown in brown. Hydrophobicity is indicated by a red shell around the atom or residue. Hydrogen bonds and distances (Å) are shown in green, and with dashed lines. This diagram was created using the ligplot software 28. The S70‐PNM covalent bond, which traps the reaction in the acyl‐intermediate state is shown in red. (B) Hydrogen bonds between the ring‐opened penicillin molecule (PNM) and enzyme residues are indicated by black dashed lines, while the structure of both are shown as yellow sticks. Nitrogen atoms are shown in blue, carbon atoms in yellow, sulfur atoms in gold, and oxygen atoms in red. (C) A rotated view of the active site shows the S70‐PNM bond that traps the reaction in the acyl‐enzyme intermediate form.

A ring‐opened penicillin molecule with a refined occupancy of 94% is covalently attached to the catalytic nucleophile Ser70 of the E166A/R274N/R276N mutant of Toho1 β‐lactamase (Fig. 2C). The carboxyl group of the ligand is involved in hydrogen bonding interactions with the sidechains of Thr235 (2.51 Å) and Ser237 (2.71 Å) while the amino sidechain groups of Asn132 (2.86 Å) and Asn104 (3.02 Å) interact with carbonyl (O16) the hydrolyzed penicillin (Fig. 2A,B). The main chain carbonyl group of Ser237 (2.92 Å) interacts with N14 of the ligand. The oxyanion hole present on the carbonyl group of the ring‐opened penicillin is stabilized by interactions with the main chain amide groups of Ser70 (2.74 Å) and Ser237 (2.89 Å; Fig. 2A). These distances are very similar to those observed in the acyl‐enzyme complex with cefotaxime 29, previously published by our group. In that structure the oxyanion hole stabilizing bond lengths are Ser70 (2.78 Å) and Ser237 (2.98 Å) 9. Shimamura *et al* 30 proposed that differences in hydrogen bond lengths in the oxyanion hole area between acyl‐intermediate structures of penicillin and acyl‐intermediate structures of cefotaxime are the reason for the lower penicillinase activity of CTX‐M type ESBL β‐lactamases, as the β‐lactam carbonyl oxygen is not well positioned in the oxyanion hole 30. However, our structures indicate that this is not the case as the observed deviations between the penicillin and cefotaxime oxyanion holes are only 0.04 Å for the Ser70 amide hydrogen bond and 0.09 Å for the Ser237 amide hydrogen bond. These negligible bond length differences show only a minor perturbation in the β‐lactam ring carbonyl group of the acyl‐intermediate structures of penicillin and acyl‐intermediate structures of cefotaxime. We, therefore, find that that β‐lactam ring carbonyl group is well positioned in the oxyanion hole for hydrolysis to occur, an essential interaction for both the acylation and deacylation reactions.

**Figure 3:**
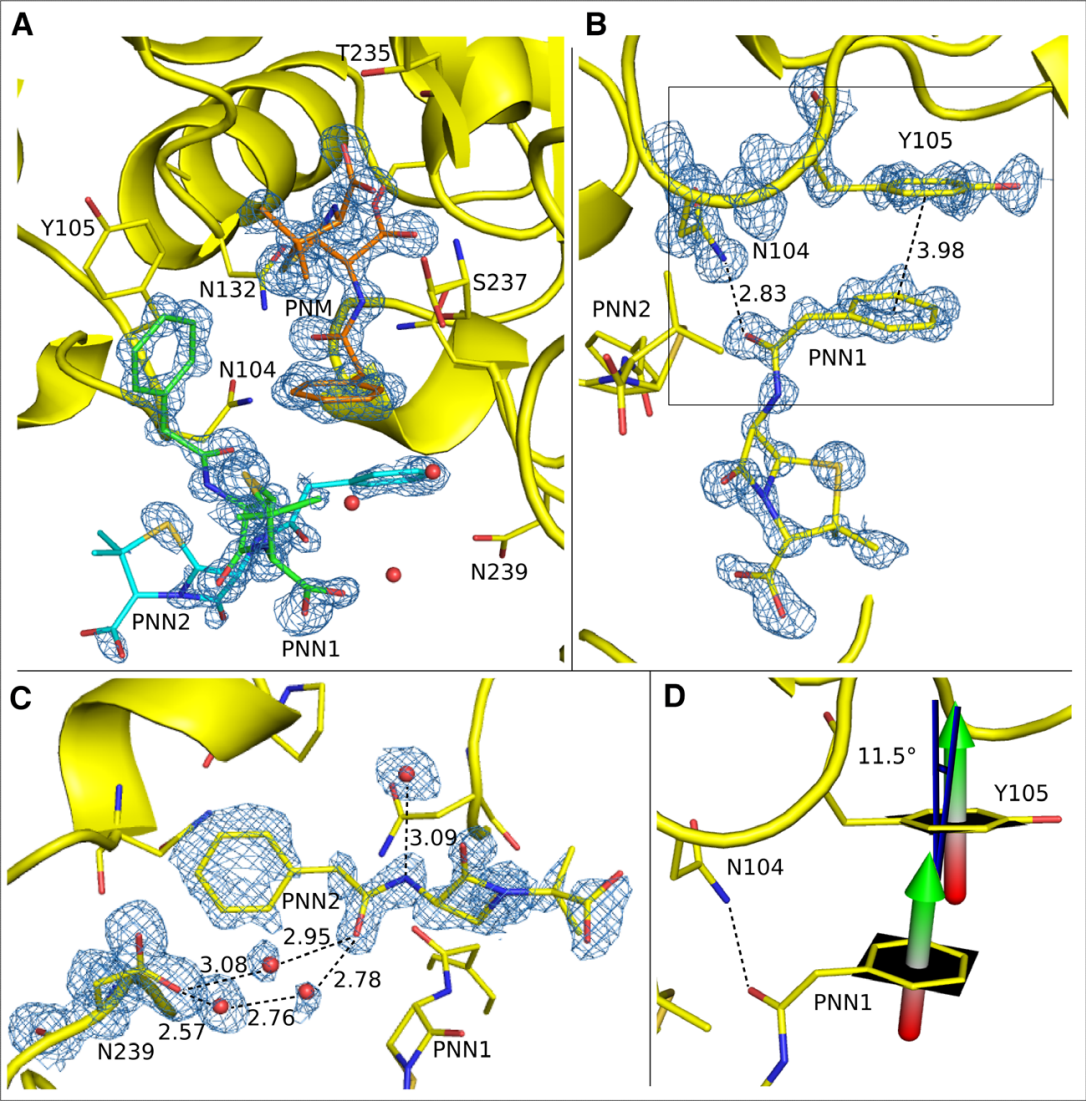
Positions and contacts of the ring‐opened penicillin molecule (PNM), and two intact penicillin molecules (PNN) in and near the active site of E166A/R274N/R276N mutant of Toho1 β‐lactamase. Interactions are indicated by dashed lines and distances are given in Å. Electron density 2F_o_‐F_c_ maps of the atoms of interest are represented by blue mesh, at a σ level of 1.5, unless indicated otherwise. (A) A ring‐opened penicillin molecule PNM is covalently bound to S70, trapping it in the active region. Two intact ring‐closed penicillin molecules PNN1 (green) and PNN2 (turquoise) occupy the region immediately outside the active site. (B) PNN1 interacts with the near active site region through a pi stacking interaction between the benzyl ring of the benzyl penicillin and Tyr105, and a hydrogen bond with the sidechain amino group of Asn104. (C) PNN2 indirectly hydrogen bonds to the sidechain carboxyl group of Asn239 through three waters. Electron density 2F_o_‐F_c_ maps are represented at a σ level of 1.0. (D) The angular difference (blue) between the two normals (green and red arrows) of the pi‐stacking ring planes (black surface) is 11.5°.

Adjacent to the active site are two intact ring‐closed penicillin molecules (PNN1 and PNN2) that are clearly visible in electron density with refined occupancy values of 67% and 58%, respectively. They are both coordinated by the enzyme just outside of the active site, through noncovalent interactions (Fig. 3A). One of these molecules, named PNN1, interacts with the sidechain of Tyr105 at the edge of the active site in a pi‐stacking interaction with the benzyl ring of the benzyl penicillin while the carbonyl group of the penicillin molecule forms a hydrogen bond with the sidechain amino group of Asn104 (2.83 Å; Fig. 3B). This pi–pi interaction has an inter‐planar distance of 3.98 Å and an inter‐planar angle of 11.5° (Fig. 3B,3D), and in combination with the Asn104 hydrogen bond lends to a well occupied PNN1 position (Fig. 3B). The second ring‐closed penicillin molecule, named PNN2, indirectly hydrogen bonds to Asn239 through three water molecules (Fig. 3C). These pi‐stacking, and hydrogen bonding interactions shed light into the ability of the enzyme to attract and line up substrate molecules, even with a PNM molecule in the active site. It should be noted that these interactions were observed in an inactive mutant. However, it is not possible to use protein crystallography with wild type β‐lactamase enzymes to study these interactions due to the enzymes high catalytic rate.

## Conclusions

Due to the low temperature cryocool technology, and consequentially low average mean anisotropic displacement parameters for all atoms in the E166A/R274N/R276N Toho1 β‐lactamase/benzylpenicillin 15 K structure, we were able to visualize for the first time three individual drug molecules interacting with the protein, at exceedingly high resolution. The first, a ring‐opened penicillin molecule is covalently attached to the catalytic nucleophile Ser70, and the close positioning of its carbonyl oxygen in the oxyanion hole provides new support to the role of the oxyanion hole in hydrolysis of this drug/enzyme pair. The second, an intact ring‐closed penicillin molecule gives evidence for substrate recognition and binding. As originally proposed by Escobar and Fink 14 the conserved class A β‐lactamase residue Tyr105 is involved in substrate recognition and binding. Later studies by Doucett *et al*. 15 used saturation mutagenesis to identify the importance of Tyr105 in substrate recognition. Our 1.10 Å, 15 K, structure of the E166A/R274N/R276N Toho1 β‐lactamase mutant shows the pi stacking interaction between Tyr105 and the benzyl ring of the benzyl penicillin substrate molecule. Tyr105 interacts with an intact ring‐closed penicillin molecule within the active site and is clearly involved in substrate recognition and binding. A second intact ring‐closed penicillin molecule is held in place near the active site, by indirect hydrogen bonding to the sidechain carboxyl group of Asn239 through three waters. Perhaps the ability of β‐lactamase to queue up substrate through pi–pi interaction and coordination through direct and indirect hydrogen bonds is partly responsible for its effectiveness. By understanding the intricacies of these enzymes not only within but also just outside of the active site, we can help drive the development of new drugs that may evade them.

## Author contributions

KLW and VGV conducted most of the experiments, SLG helped with experimental setup and data collection at APS, PSL, JBC and LC analyzed the results, and wrote most of the paper.
